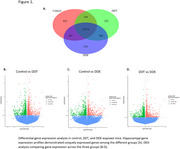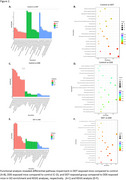# Transcriptomic Analysis of the effects of DDT, and its metabolite, DDE, on Alzheimer's pathology in 5xFAD mice

**DOI:** 10.1002/alz70855_106994

**Published:** 2025-12-24

**Authors:** Arturo J Barahona, Isha Mhatre‐Winters, Ferass M Sammoura, Yoonhee Han, Jason R Richardson

**Affiliations:** ^1^ Robert Stempel College of Public Health and Social Work, Florida International University, Miami, FL, USA; ^2^ Isakson Center for Neurological Disease Research, College of Veterinary Medicine, University of Georgia, Athens, GA, USA; ^3^ Florida International University, Miami, FL, USA

## Abstract

**Background:**

Alzheimer's disease (AD) is the most common neurodegenerative disease, with increasing evidence implicating environmental factors in its etiology. We previously reported that DDT exacerbates amyloid pathology in the 3xTG‐AD mouse model and that AD patients have significantly higher serum levels of DDE, the long‐lived metabolite of DDT that is thought to be non‐neurotoxic. However, whether DDE directly contributes to AD pathology remains unclear.

**Method:**

Male 5xFAD mice, 6 weeks of age, were exposed to 3 mg/kg of DDT, DDE, or corn oil every 3 days for 90 days. At 4.5 months of age, mice were sacrificed one day after behavioral assessment and brain tissue was dissected for RNA‐sequencing. Count data was generated using the STAR protocol and analyzed using the DESeq2 pipeline. Moreover, multiplexed gene expression of pooled samples was measured using nCounter for a more targeted approach.

**Result:**

RNA‐sequencing analysis identified 223, 252, and 213 uniquely expressed genes within control 5xFAD, DDT, and DDE‐exposed hippocampal samples, respectively. Differential gene expression analysis demonstrated that, while DDT and DDE both significantly alter a broad range of genes compared to control 5xFAD, DDT and DDE target distinct biological pathways. Gene ontology enrichment analysis revealed that DDT‐exposed mice had alterations of cellular components relating to the extracellular matrix, while DDE‐treated mice were had more pathways altered related to glial cell differentiation. KEGG analysis indicated significant impairment of the PI3K‐Akt signaling pathway in DDT‐treated mice compared to control 5xFAD, while ECM‐receptor interactions were significantly altered compared to DDE‐exposed groups. Additionally, neuronal markers, measured using nCounter were differentially expressed, with glutamatergic and dopaminergic synapse pathways affected in both exposure groups.

**Conclusion:**

Overall, these findings demonstrate that exposure of 5xFAD mice to DDT and DDE differentially affect AD‐related pathways and neurodegeneration. Importantly, they provide mechanistic evidence of DDE's neurotoxicity, reinforcing epidemiological findings and challenging the assumption that DDE is biologically inert in the brain.